# Supplementary material for: Downregulation of Mirlet7 miRNA family promotes Tc17 differentiation and emphysema via de-repression of RORγt
Source: eLife. 2024 May 9;13:RP92879. doi: 10.7554/eLife.92879 (PMC11081633; doi:10.7554/eLife.92879)
Supplement: Supplementary file 1. — Primer names and sequences are indicated above. [file elife-92879-supp1.docx]

**Genotyping Primers**

| **Name** | **Sequence** | **Description** |
| --- | --- | --- |
| BC-lox2-F | 5'-GGACATGAGATCGCCAACCA-3' | *Mirlet7bc2*-cluster floxed allele Forward primer for genotyping |
| BC-lox2-R | 5'-TGGAAGCCAGTACTGTGCTC-3' | *Mirlet7bc2*-cluster floxed allele Reverse primer for genotyping |
| AFD-lox2-F | 5’-GTTTTCTGAGGTGTGGGAGGTA-3’ | *Mirlet7afd*-cluster floxed allele Forward primer for genotyping |
| AFD-lox2-R | 5’-AGTGGGATAGAAGGATCTCAGG-3’ | *Mirlet7afd*-cluster floxed allele Reverse primer for genotyping |

**Dharmacon Duplexes**

| **Name** | **Sequence** |
| --- | --- |
| *hsa-let-7b-5p* | 5'-UGAGGUAGUAGGUUGUGUGGUU-3' |
| Control (*cel-miR-67-3p*) | 5'-UCACAACCUCCUAGAAAGAGUAGA-3' |
