# Supplementary material for: Downregulation of Mirlet7 miRNA family promotes Tc17 differentiation and emphysema via de-repression of RORγt
Source: eLife. 2024 May 9;13:RP92879. doi: 10.7554/eLife.92879 (PMC11081633; doi:10.7554/eLife.92879)
Supplement: Supplementary file 2. — Mean ± standard deviation is shown for age, FEV1 %, FEV1/FVC %. Abbreviations: FEV1 (forced expiratory volume in 1 s). FEV1/FVC (forced expiratory volume/forced vital capacity). [file elife-92879-supp2.docx]

| Emphysema  Severity Score | = 0 | = 1 | = 2 | = 3 |
| --- | --- | --- | --- | --- |
|  |  |  |  |  |
| Sex, n (%) |  |  |  |  |
| Male | 2 (100) | 5 (100) | 5 (100) | 6 (85.7) |
| Female | 0 (0) | 0 (0) | 0 (0) | 1 (14.3) |
| Age | 74.0 ± 4.2 | 66.0 ± 7.6 | 65.2 ± 4.6 | 67.1 ± 3.9 |
| Race, n (%) |  |  |  |  |
| Caucasian | 2 (100) | 5 (100) | 4 (80) | 5 (71.4) |
| African American | 0 (0) | 0 (0) | 1 (20) | 2 (28.6) |
| Current Smoker, n (%) |  |  |  |  |
| Yes | 0 (0) | 2 (40) | 3 (60) | 4 (57.1) |
| No | 2 (100) | 3 (60) | 2 (40) | 3 (42.9) |
| FEV_1_ % | 57.0 ± 17.0 | 68.8 ± 12.3 | 74.2 ± 13.9 | 80.7 ± 9.5 |
| FEV_1_/FVC % | 68.5 ± 12.0 | 66.6 ± 5.7 | 70.3 ± 9.4 | 60.3 ± 6.7 |
